# Mind the Metabolic Gap: Bridging Migraine and Alzheimer's disease through Brain Insulin Resistance

**DOI:** 10.14336/AD.2024.0351

**Published:** 2024-06-16

**Authors:** Lorenzo Del Moro, Elenamaria Pirovano, Eugenia Rota

**Affiliations:** ^1^Personalized Medicine, Asthma and Allergy, IRCCS Humanitas Research Hospital, Rozzano (MI), Italy.; ^2^Center for Research in Medical Pharmacology, University of Insubria, Varese, Italy.; ^3^Neurology Unit, San Giacomo Hospital, Novi Ligure, ASL AL, Italy.; ^4^Department of Biomedical Sciences, Humanitas University, Pieve Emanuele, Milan, Italy

**Keywords:** metabolism, migraine, Alzheimer's disease, brain insulin resistance, glucose

## Abstract

Brain insulin resistance has recently been described as a metabolic abnormality of brain glucose homeostasis that has been proven to downregulate insulin receptors, both in astrocytes and neurons, triggering a reduction in glucose uptake and glycogen synthesis. This condition may generate a mismatch between brain’s energy reserve and expenditure, ??mainly during high metabolic demand, which could be involved in the chronification of migraine and, in the long run, at least in certain subsets of patients, in the prodromic phase of Alzheimer’s disease, along a putative metabolic physiopathological continuum. Indeed, the persistent disruption of glucose homeostasis and energy supply to neurons may eventually impair protein folding, an energy-requiring process, promoting pathological changes in Alzheimer's disease, such as amyloid-β deposition and tau hyperphosphorylation. Hopefully, the “neuroenergetic hypothesis” presented herein will provide further insight on there being a conceivable metabolic bridge between chronic migraine and Alzheimer’s disease, elucidating novel potential targets for the prophylactic treatment of both diseases.

## Introduction

Migraine and Alzheimer's disease (AD) remain two major public health issues. Indeed, migraine and other primary headache disorders are the second leading causes of disability worldwide, according to The World Health Organization [1]. Notably, migraine is the leading cause of disability in the under-50s [2], affecting about 14% of the world's population [3]. Although migraine is generally an episodic disorder, it may evolve over time into a chronic condition, with an average annual progression rate of 3% [4]. AD is the most common form of dementia in developed countries and its prevalence is on the increase, due to population aging [5].

Indeed, the Global Burden of Disease 2019 Dementia Forecasting Collaborators estimated that the number of people with dementia would have increased from 57.4 million cases globally in 2019 to 152.8 million cases in 2050 worldwide [6]. Despite recent therapeutic advances [7], there are still unmet needs in migraine preventive treatment [8]. Likewise, halting the progression of AD currently remains a challenge [9]. Although the novel anti-amyloid antibody treatments have reached the objective of promoting amyloid-β (Aβ) clearance and slowing down AD progression over several months, their efficacy is only moderate. Indeed, there are still numerous hurdles to overcome to improve their long-term efficacy, safety and accessibility [10-12]. Therefore, research has focused on other pathophysiological factors that might play a role in the complex aetiology of AD, such as a decrease in the brain energy metabolism [13, 14].

**Figure 1.**
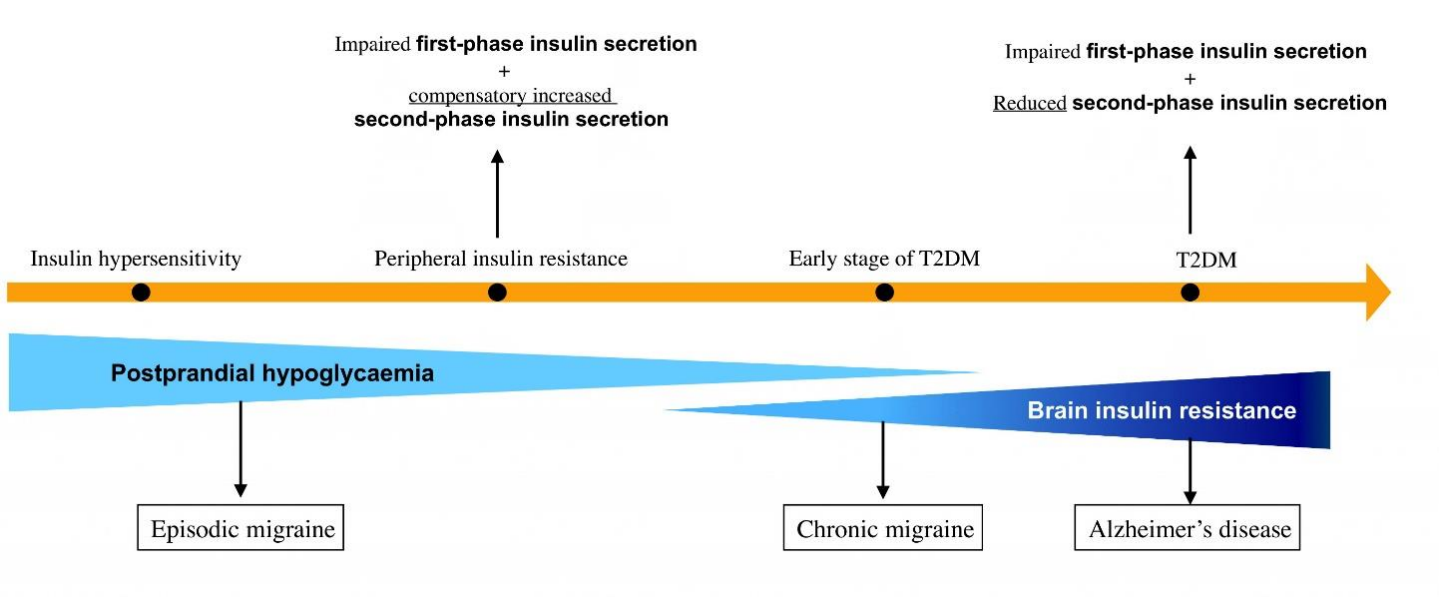
**The “Neuroenergetic hypothesis”: a metabolic bridge between migraine and Alzheimer’s disease (AD)**. This illustrates the Neuroenergetic Hypothesis that we first described elsewhere [15]. Herein we focused on the “extended Neuroenergetic Hypothesis” as to there being a metabolic bridge between chronic migraine and AD. Conditions that are connected by a continuum of time and pathophysiology are marked by an orange arrow, i.e., insulin hypersensitivity, insulin resistance, early stage of T2DM and T2DM. Both insulin hypersensitivity and peripheral insulin resistance can lead to postprandial hypoglycaemia through different mechanisms. Postprandial hypoglycaemia has been identified as a major contributor to cerebral energy deficiency that underlies episodic migraine. The worsening of glucose metabolism, evidenced by the orange arrow, may extend to the brain over time, leading to brain insulin resistance. Brain insulin resistance generates a chronic mismatch between the energy reserve of the brain and functional expenditure, which is involved in the chronification of migraine and, in the long run, at least in certain subsets of patients, in the prodromic phase of AD, along a putative metabolic physiopathological continuum. T2DM, type 2 diabetes mellitus.

After having reviewed the current literature on the metabolic aspects of migraine pathophysiology, we proposed a "neuroenergetic hypothesis” of migraine [15] (Fig. 1).

We assumed that an *energy deficit* (a mismatch between the brain’s energy reserve and workload), caused by an altered glucose and insulin metabolism in the brain, i.e., the condition of *brain insulin resistance*, may be a pivotal mechanism in the pathophysiology of migraine, promoting its chronification.

## Background of the “Neuroenergetic hypothesis”

In 2017, Blonz first proposed a “Neuroenergetic hypothesis” for AD [13] which, interestingly, may overlap with some pathophysiological alterations in migraine. He hypothesized that the decreased availability of metabolizable energy resources in the central nervous system is a key factor in AD pathogenesis, mainly as a result of an age-related decline in the ability of glucose to cross the blood-brain barrier.

After which, Zulfiqar et al. [16] revised the "Neuroenergetic hypothesis" and proposed that AD may be underpinned by “a novel” pathophysiological mechanism. They reported that, in their opinion, cerebral glucose hypometabolism is an early event in AD, caused by a deficit in the support of neuronal physiological needs, mainly due to an imbalanced neuron-astrocyte lactate shuttle. This would imply that astrocytes play a key role in this revised "Neuroenergetic hypothesis" of AD pathophysiology [16]. This seems to be in line with what was previously reported by other authors in 2015, i.e., that astrocyte hypertrophy and lesions occur early in AD progression [17].

Therefore, the "Neuroenergetic hypothesis" seems to be an appealing theoretical frame, accounting for some brain metabolic abnormalities shared by migraine and AD and providing further insight into a putative metabolic bridge between chronic migraine (CM) and AD.

## Migraine and Alzheimer’s disease may have overlapping pathophysiological mechanisms

**Figure 2.**
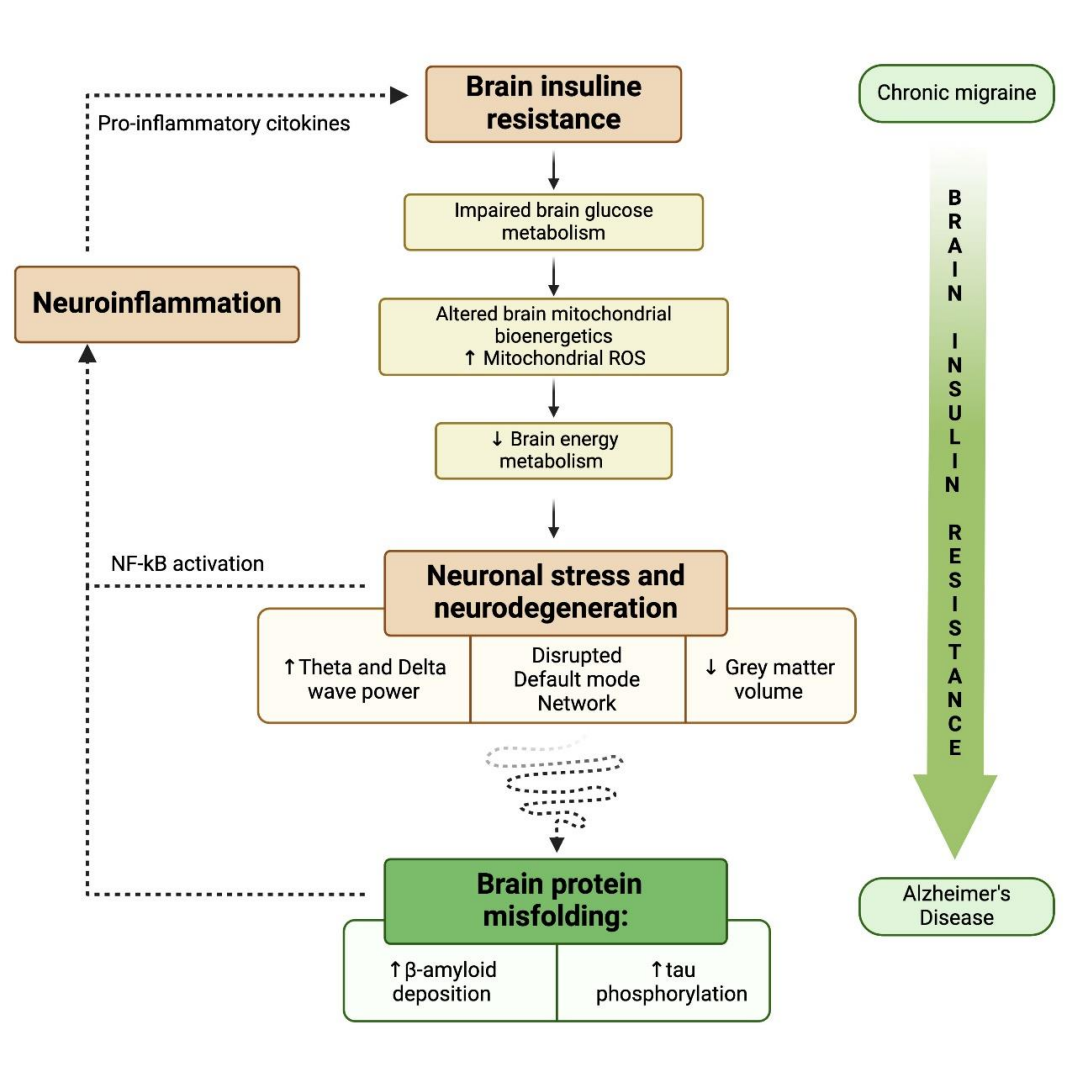
**From migraine to Alzheimer’s Disease (AD): the metabolic physiopathological continuum of the “Neuroenergetic hypothesis”**. Brain insulin resistance is the metabolic alteration underlying the common pathophysiological alterations between chronic migraine (CM) and AD, i.e., impaired brain glucose metabolism and altered brain mitochondrial bioenergetics - leading to an overproduction of mitochondrial reactive oxygen species - which generate a reduction in the brain energy metabolism, leading to neuronal stress and subsequent neuronal degeneration, detectable as a reduction in grey matter volume, a disrupted default mode network connectivity and the increased theta and delta activity observed at EEG, shared by CM and AD. In the long run, brain insulin resistance and the related energy deficiency might favour the pathological changes involved in AD, promoting a shift towards the amyloidogenic cascade and increasing tau hyperphosphorylation. The shaded green arrow to the right of the figure illustrates that increased brain insulin resistance sustains the altered metabolic pathway and the progression from CM to AD.

This scoping review focuses on the fact that, as aforementioned, there are intriguing similarities between the pathophysiology of CM and AD: brain insulin resistance, impaired brain glucose metabolism, an alteration in brain mitochondrial bioenergetics and neuroinflammation. These are likely to be common pathophysiological alterations shared by these two pathological conditions and may well underlie the reduction in grey matter volume in specific areas, the disrupted default mode network connectivity observed at neuroimaging and the increased theta and delta activity evidenced on EEG in both diseases (Fig. 2).

Indeed, in the long-run, the persistent disruption of the glucose homeostasis may impair protein folding, which is an energy-requiring process essential for brain tissue turnover and functionality. This might be one of the mechanisms, promoting a shift towards the amyloidogenic pathway and tau phosphorylation, in the complex pathophysiology underlying AD pathological changes [18] (Fig. 2).

Therefore, in our hypothesis we propose that brain insulin resistance could be considered a “metabolic bridge” between CM and AD, in a sort of pathophysiological continuum. Recent epidemiological studies also support this hypothesis, as they report a positive association between migraine and the subsequent development of dementia, where migraine with aura in early life is associated with a two-fold increased risk of dementia and a four-fold increased risk of AD [19, 20].

The last paragraph discusses potential treatment options, targeting the mechanisms involved in such a pathophysiological hypothesis, these include: the traditional Mediterranean Diet, regular aerobic exercise and Mind-Body Interventions (MBI). Although the “Neuroenergetic Hypothesis” as a potential therapeutic target is still a major concern, these sustainable long-term options could significantly reduce the frequency and intensity of migraine attacks and, hopefully, also play a role in preventing or, at least, delaying, the onset of some types of dementias, in particular AD. Further clinical studies are required to prove this hypothesis.

## Epidemiological evidence on a putative correlation between migraine and dementia

Although the question of the relationship between migraine and dementia remains controversial, in particular how migraine and AD may influence each other, there is an increasing body of evidence (Table 1) as to a positive association between these two conditions. Recent data from observational and investigational studies (Table 1) suggest that migraine - especially migraine with aura - may be a risk factor for dementia, mainly AD, with a weaker positive association also for vascular dementia (VaD).

**Table 1:** Summary of the studies investigating the epidemiological relationship between migraine and dementia.

First author	Year	Method	Country	Observations
Cermelli A [21]	2023	Systematic review and meta-analysis	Not applicable	Migraine was associated with both a moderate increased risk of all-cause dementia (OR = 1,26; p = 0,00; 95% CI: 1,13-1,40) as well as a moderate increased risk of Alzheimer's disease (AD) (OR = 2,00; p = 0,00; 95% CI: 1,46-2,75).
Qu H [22]	2022	Meta-Analysis of Cohort Studies	Not applicable	Types of dementia considered: all-cause dementia, AD, vascular dementia (VaD).A history of migraine is associated with a higher risk of dementia (OR = 1.32; 95% CI: 1.13-1.40; I2 = 75.6%, P < 0.001), but the risk is lower than that of non-migraine headache patients.
Wang L [23]	2022	Meta-Analysis of Cohort Studies	Not applicable	Pooled analysis showed that migraine was associated with increased risk of all-cause dementia (RR: 1.34, 95% CI: 1.13-1.59) and AD (RR: 2.49, 95% CI: 1.16-5.32). However, we did not find any association between migraine and risk of VaD (RR: 1.51, 95% CI: 0.77-2.96).
Kim SJ [24]	2022	Nationwide Retrospective Cohort Study	South Korea	Patients with migraine had a 1.18 (adjusted hazard ratio [aHR], 1.18; 95% CI, 1.12-1.24), 1.21 (aHR, 1.21; 95% CI, 1.10-1.32), and 1.18 (aHR, 1.18; 95% CI, 1.13-1.24) times higher risk of developing AD, VaD and all-cause dementia.
Hurh K [25]	2022	Nationwide Retrospective Cohort Study.	South Korea	Patients with migraine had a 1.30 (hazard ratio [HR], 1.30; 95% CI, 1.25-1.35), 1.29 (HR, 1.29; 95% CI, 1.23-1.35), 1.35 (HR, 1.35; 95% CI, 1.19-1.54), 1.36 (HR, 1.36; 95% CI, 1.00-1.83), and 1.30 (HR, 1.30; 95% CI, 1.17-1.45) times higher risk of developing all-cause dementia, AD, VaD, mixed or other specified dementias, and unspecified dementia than their matched controls, respectively.
Gu L [26]	2022	Meta-Analysis	Not applicable	The study showed no significant association between migraine without aura and risk of dementia with a random effects model (OR/RR = 1.03, 95% CI 0.89 to 1.19, I2 = 0.0%, p = 0.453). The study showed significant associations between migraine and risk of VaD (OR/RR = 1.84, 95% CI 1.18 to 2.88, I2 = 0.0%, p = 0.423) and AD (OR/RR = 2.60, 95% CI 1.51 to 4.48, I2 = 43.8%, p = 0.169) with random effects models.
Lee HJ [27]	2021	Nationwide Retrospective Cohort Study	South Korea	Patients with migraine had a significantly higher incidence of AD (adjusted HR = 1.31, 95% CI, 1.08-1.58), but not VaD, than those without migraine.
Islamoska S [19]	2020	Longitudinal population-based register study.62578 individuals, 10857 with migraine.Follow-up 6.9 years.	Denmark	Types of dementia considered: unspecified dementia, AD, VaD, frontotemporal dementia, and Lewy body dementia. They were not evaluated individually.207 individuals with migraine developed dementia. Individuals without aura had a 19% higher rate of dementia, and individuals with aura had a two-fold higher rate of dementia, compared with individuals without migraine.
Kostev K [28]	2019	Retrospective cohort study. 7454 individuals from 67 general practices inthe UK	United Kingdom	Types of dementia considered: VaD, AD, unspecified dementia.They observed - only in women - a positive significant association between migraine diagnoses and all-cause dementia (hazard-ratio [HR] = 1.65) as well as AD (HR = 2.27).
Morton RE [20]	2019	Prospective cohort study. 679 community-dwelling participants 65+ years, follow up 5 years.	Canada	A history of migraines was significantly associated with both all-cause dementia (odds ratio [OR]=2.97; 95% confidence interval [CI]=1.25-6.61) and AD (OR=4.22; 95% CI=1.59-10.42). Although no significant association was found between migraine and vascular dementia.
Lee SY [29]	2019	Retrospective cohort study. 11438 dementia participants, 45752 controls	Korea	Dementia was defined as diagnoses of AD or dementia in AD.7.7% of patients in the dementia group and 6.3% of those in the control group had a history of migraine.The crude and adjusted odds ratios for migraine with dementia was 1.22 and 1.13, respectively.
Hagen K[30]	2014	A prospective population-based study. 51,383 participants from the Nord-Trøndelag Health Study	Norway	There was a significant interaction between age and any headache regarding VaD (p < 0.0001): In subsequent analyses stratifying by age, any headache increased the risk of VaD more among individuals 75 years of age at baseline (HR ¼ 3.0; 95% CI 1.3-6.7, p ¼ 0.007) than among those.They observed a positive association between VaD diagnoses and all types of migraine (hazard-ratio [HR] = 2.9; 95% confidence interval [CI]= 1.3-6.6). This was more marked for migraine on 15 days/month (HR = 9.1 (2.2-40.1), p = 0.003) than for nonmigrainous headache on 15 days/month (HR = 3.2 (1.0-10.3), p = 0.057).No association was found between headache and AD. However, the association between AD and migraine has not been investigated.
Chuang CS [31]	2013	Retrospective cohort study.Data from the National Health Insurance Research database in Taiwan	Taiwan	Types of dementia considered: AD, senile dementia, dementia in conditions classified elsewhere (e.g., dementia of the Alzheimer's type). They were not evaluated individually.After adjusting the covariates, migraine patients had a 1.33-fold higher risk of developing dementia, compared with individuals without migraine. Young adults have a higher association between migraine and dementia than older adults.

This association may arise from the well-known vascular comorbidity of migraine. However, it is still a question of debate as to whether migraine should be considered a true “vascular disease” or if the comorbidity between migraine and cerebrovascular disease may have underlying shared risk factors or pathophysiological mechanisms [32, 33].

Three meta-analyses on the association between migraine and dementia were published in 2022. Qu et al. reported that a history of migraine is associated with a higher risk of all-cause dementia, but the risk is lower than that of non-migraine headache patients [22]. Wang et al. demonstrated that migraine was associated with an increased risk of all-cause dementia, especially AD [23]. Gu et al. observed significant associations between migraine with aura and risk of VaD and AD and that the association was stronger for AD [26].

In the same year, two Nationwide Retrospective Cohort Studies, performed in South Korea, also reported that patients with migraine run a higher risk of subsequently developing dementia, in particular, VaD [24, 25]. Moreover, in 2023 a systematic review and meta-analysis reported that migraine was associated with both a 26% increased risk of all-cause dementia as well as a two-fold increased risk of AD [21]. Therefore, although the epidemiological evidence linking migraine with a higher risk of dementia and AD is compelling, further studies are still required to understand the nature of this association and whether this implies a direct causative relationship or shared risk factors.

Our scoping review fits into this framework, as it proposes a pathophysiological hypothesis that may explain at least some of the mechanisms involved in this association, which still remain partly unknown.

### Insulin resistance and the brain

Insulin resistance is commonly characterized as a decreased sensitivity of bodily tissues to the action of insulin [34]. It may be defined as a subnormal physiological response of target tissue to insulin stimulation [35]. Similarly, brain insulin resistance is the inability of brain cells to respond to insulin [36]. Systemic and cerebral insulin resistance may have a strong correlation, in as much as systemic insulin resistance, in patients with type 2 diabetes (T2DM), may lead to brain insulin resistance and brain dysfunction, whereas aberrant insulin signalling in the brain may have systemic repercussions that affect metabolic regulation [37-39]. Although the question of the relationship between brain and peripheral insulin resistance is currently under debate. The two conditions are not always interlinked, and it remains to be confirmed whether peripheral and central insulin resistance are able to exist independently [40]. Moreover, at present, there is no internationally accepted criteria for the identification of a neurophysiological or neuroimaging response as a marker of brain insulin resistance [41]. The methods to investigate brain insulin resistance have been reviewed elsewhere [40].

Various mechanisms may underlie the diminished response to insulin, such as the downregulation of insulin receptors, the inability of insulin receptors to bind insulin and/or the aberrant activation of the insulin signalling cascade [37]. At a cellular level, this abnormality may manifest itself as an impaired neurotransmitter release, altered receptor regulation in neurons and glial cells and/or a dysfunction in the processes that are most directly related to insulin metabolism and glucose homeostasis, such as glucose uptake in neurons and inflammatory responses to insulin [42, 43]. The role of insulin resistance in the context of the “Neuroenergetic hypothesis” in migraine pathophysiology and chronification was recently assessed in a review by Del Moro et al. [15].Growing evidence suggests that insulin resistance is a pivotal pathophysiological mechanism also in AD, which is emerging as ‘‘type 3 diabetes’’, in agreement with Steen et al.’s hypothesis [39]. Other authors [18, 44, 45] further developed this hypothesis, reporting that oxidative stress, impaired glucose metabolism and tau hyperphosphorylation and Aβ deposition were all linked to perturbation in insulin/insulin-like growth factor signaling.

Indeed, anti-diabetic drugs, such as metformin, intranasal insulin, incretins, SGLT2 inhibitors, PPAR-γ agonists and DPP4 inhibitors are now being investigated in the context of AD treatment and prevention. Most of these drugs have provided some promising results in clinical trials; however, additional research is required to confirm their therapeutic potential [46, 47].

To date, no randomized controlled trial (RCT) that evaluates the safety and efficacy of anti-diabetes drugs in the treatment of chronic migraine has been published.

### Decreased brain glucose metabolism

The results of positron emission tomography (PET) studies and voxel-based statistical parametric mapping analysis of (18)F-fluorodeoxyglucose-PET report functional neuroimaging evidence of a decreased cerebral glucose metabolism in migraine patients, especially in CM [48-50].

Notably, certain brain regions in AD (Table 2) were identified as being particularly vulnerable to hypometabolism [79, 80], a reduced glucose metabolic rate [60, 62], a reduced cortical thickness, volume loss [64], atrophy [43, 78] and amyloid deposition [87]. These areas were named *AD-vulnerable brain regions* [64, 71, 81, 87], and include the parietal cortex, the posterior cingulate cortex, the temporal gyrus, the temporal pole, the medial temporal lobe (parahippocampal gyrus, hippocampus, amygdala, entorhinal cortex), the prefrontal cortex and the superior and middle frontal gyrus (Table 2).

**Table 2:** Comparison between brain areas affected by reduced glucose metabolism, volume and energy metabolism in insulin resistance (IR), Alzheimer’s disease (AD-vulnerable brain regions) and migraine (migraine-vulnerable brain regions).

Brain areas	Reduced regional cerebral glucose metabolism in subjects with insulin resistance	Reduced regional cerebral glucose metabolism in migraine subjects	Reduced regional cerebral energy metabolismin migraine subjects ***	Reduced regional cerebral volume in migraine subjects	Alzheimer’s disease - vulnerable brain regions
The insular lobe		Insular lobe: CM [50]Insular cortex: EM [48], CM [49]		Insular lobe: † [51], [52]Insular cortex: *CM [53]	
The parietal lobe	Parietal lobe: [54] lateralParietal cortex: †††† [55] leftBrodmann areas 7 and 40: [56]	Parietal cortex: CM [49]	↓ PCr/Pi ¯ [57]↓ [Mg^+2^] ¯ [58]↓ PCr/Pi ¯ [59]	Parietal lobe: CM [53]Parietal operculum: † CM [51] left	Parietal lobe: [60-62]Parietal cortex: [43]Parieto-temporal cortex: [63]Inferior parietal cortex: [64]
The anterior cingulate cortex		Anterior cingulate cortex: CM [49], [48]		Anterior cingulate cortex: ** EM e CM [65], † CM [51], CM [66], [67] right, [52]	
The posterior cingulate cortex	Posterior cingulate cortex: [56]	Posterior cingulate cortex: EM [48]			Posterior cingulate cortex: [60-64, 68]
The temporal lobe	Temporal lobe: [54]Middle temporal cortex: †††† [55] leftTemporal/angular gyri (Brodmann area 39): [56]	Inferior temporal, temporal pole, right-banks superior temporal sulcus: CM [50]	↓ PCr/Pi ¯ [57]↓ [Mg^+2^ ] ¯ [58]↓ PCr/Pi ¯ [59]	Temporal pole, superior temporal lobe: CM [66] Left superior temporal gyrus, right fusiform gyrus, right middle temporal gyrus: CM [69]Superior temporal sulcus left, inferior temporal gyrus left : CM [69]	Temporal lobe: [43, 60]Temporal cortex: [64, 68] lateral, [70]Inferior temporal gyrus: [71]Temporopolar cortex: [64]
The prefrontal cortex	Prefrontal cortex:[54]Anterior and inferior prefrontal cortices (Brodmann areas 10, 45, 47): [56]	Prefrontal cortex: EM [48] leftPars triangularis (Brodmann areas 44): CM [50] Orbitofrontal cortex (Brodmann area 10, 11 and 47)****: CM [49]Orbitofrontal (Brodman area 47)††: CM [50] left		Inferior frontal gyri (Brodmann areas 44, 45, 47)*****:CM [51]†, EM [72], [67]Dorsolateral prefrontal cortex (Brodmann areas 46 and 9): EM [65, 73]Pars triangularis (Brodmann areas 44): CM [66]Lateral orbital frontal cortex (Brodman area 47)††: CM [69, 74, 75] left, ††† CM [76]Medial orbital frontal gyrus: CM [66]	Prefrontal Cortex [43]Inferior frontal cortex (Brodmann areas 44, 45, 47)*****: [64]
The frontal lobe	Superior frontal gyrus: †††† [55] right Middle frontal gyrus: †††† [55]	Superior frontal gyrus: CM [50] leftFrontal pole: CM [50] right Precentral gyrus: CM [50] right	↓ PCr/Pi ¯ [57]↓ Pi/Tp ¯ [57]↓ [Mg^+2^] ¯ [58]↓PCr/Pi ¯ [59]	Medial frontal lobes: ††† CM [76]Superior frontal gyrus: CM [75]Middle frontal gyrus: [67], CM [53] caudal Precentral gyrus: CM [53, 69, 72], [67] rightRight frontal pole: CM [74],††† [76]	Frontal lobes: [60, 61, 63]Superior frontal gyrus: [68]Middle frontal gyrus: [68, 71]
The hippocampus	Hippocampus: [55]			Hippocampus: CM [65, 77] leftEntorhinal cortex: CM [66]Parahippocampal gyrus: [67] leftParahippocampus:EM [72]	Hippocampus: [43, 60, 62, 64, 68, 78-81]Entorhinal cortex:[64, 68, 81]Parahippocampal gyrus:[68]
The amygdala	Amygdala: [55]			Amygdala: † [51, 82] left	Amygdala: [61, 68, 78, 81]
The occipital lobe			↓ PCr/Pi ¯, ↓ [Mg^+2^ ] ¯ [82]		

**Table:** 

EM	episodic migraine
CM	chronic migraine
PCr	phosphocreatine
Pi	inorganic phosphate
TP	total phosphorus signal
PCr/Pi, PCr/ATP	a reduced ratio indicates energy deficit
ATP	adenosine triphosphate
In bold	brain areas affected by reduced glucose metabolism and shared by insulin resistance, migraine and AD subjects.
*	Lai KL et al enrolled patients with CM without medication overuse headache, major depression or prior preventive treatment.
**	A higher headache frequency was associated with smaller grey matter volume in the anterior cingulate cortex and hippocampus in EM and CM
***	According to current literature, most studies have chosen the occipital cortex as the region of interest, as aura, most commonly with visual symptoms, is attributed to this area in patients suffering from this type of migraine [83]
****	The orbitofrontal cortex includes the Brodmann areas 10, 11 and 47 [84]
*****	The inferior frontal gyrus includes the Brodmann areas 44, 45 and 47 [85]
†	In comparing the brains of CM patients with EM patients, Valfrè et al reported that CM patients had significant grey matter reductions in these areas.
††	The lateral orbital gyrus includes the Brodmann area 47 [86] *Mackey, Sott; Petrides, Michael (2006). "Chapter 2: The orbitofrontal cortex: sulcal and gyral morphology and architecture". In Zald, David H.; Rauch, Scott (eds.). The Orbitofrontal Cortex. New York: Oxford University Press. p. 34*
†††	Chronic migraine patients had smaller frontal regions than episodic migraine patients.
††††	This study on young women with Polycystic Ovary Syndrome reported a direct association between mild insulin resistance and brain glucose hypometabolism, which was independent of overweight or obesity.

Neuroimaging studies on migraine also revealed structural and functional changes in certain brain regions of sufferers - therefore, in our opinion, these could be named, “*migraine-vulnerable brain regions*”, also suggesting an association between attack frequency and the degree of abnormalities [52].

**Figure 3.**
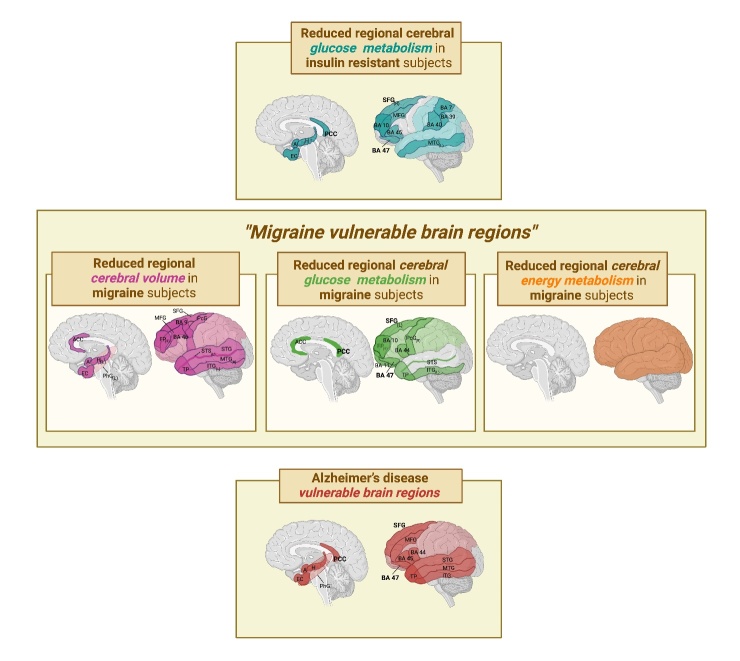
**Graphic representation of brain areas affected by reduced glucose metabolism, volume and energy metabolism in insulin resistance, migraine (i.e., “*migraine-vulnerable brain regions*”) and Alzheimer’s disease (i.e., *AD-vulnerable brain regions*)**. Insulin resistance, migraine and AD subjects share three brain areas (bold) affected by reduced glucose metabolism (i.e., the superior frontal gyrus, the posterior cingulate cortex and the Brodmann area 47). Chronic migraine and AD subjects share several brain areas affected by volume loss; they include the medial temporal lobe (parahippocampal gyrus, hippocampus, amygdala, entorhinal cortex), the superior and middle frontal gyrus, the inferior frontal cortex (i.e., the Brodmann areas 44, 45, 47), the parietal cortex, the temporal gyrus and the temporal pole. (L), left; (R), right; A, amygdala; H, hippocampus; PCC, posterior cingulate cortex; BA, Brodmann area; SFG, superior frontal gyrus; MFG, middle frontal gyrus; MTG, middle temporal gyrus; ACC, anterior cingulate cortex; EC, enthorinal cortex; PhG, parahippocampal gyrus; FP, frontal pole; PcG, precentral gyrus; SFG, superior frontal gyrus; TP, temporal pole; STG, superior temporal gyrus; ITG, inferior temporal gyrus; STS, superior temporal sulcus.

Interestingly, several “*migraine-vulnerable brain regions*” match the *AD-vulnerable brain regions* (Fig. 3, Table 2). Noteworthy is the fact that the superior frontal gyrus, the posterior cingulate cortex and the Brodmann area 47, part of the prefrontal cortex [85], are affected by glucose hypometabolism in insulin resistance, migraine and AD subjects (Fig. 3, Table 2), suggesting a shared metabolic alteration between the three conditions. Moreover, CM and AD subjects share several brain areas affected by volume loss; they include the medial temporal lobe (parahippocampal gyrus, hippocampus, amygdala, entorhinal cortex), the superior and middle frontal gyrus, the inferior frontal cortex (i.e., the Brodmann areas 44, 45, 47 [85]), the parietal cortex, the temporal gyrus and the temporal pole (Fig. 3, Table 2). Indeed, the energy deficit, most likely promoted by insulin resistance, may lead to neuronal dysfunction and, over time, neurodegeneration and lobe atrophy [88].

Indeed, our “Neuroenergetic Hypothesis” proposes that brain insulin resistance, glucose hypometabolism, and energy deficit are related pivotal factors that contribute to the neuronal stress involved in migraine attack chronification, and, subsequently, if they persist over time, in the prodromal stage of AD, at least in some subgroups of patients, along a pathophysiological continuum (Fig. 2).

This is supported by other evidence:

a. The glucose transporter type 4 (GLUT4), which is insulin-sensitive [15], is expressed by neurons and astrocytes in some brain regions which are particularly responsive to insulin and related to memory, learning, emotional and cognitive functions; these include the hippocampus, amygdala and a vast area of the cerebral cortex (i.e., posterior cingulate cortex, temporo-parietal cortex, frontal and prefrontal cortex) [37, 89-92]. This suggests that the insulin signalling pathway may play a key role in glucose utilization in these areas [90]. Noteworthy is the fact that all these areas are affected in insulin resistance, migraine and AD (Fig. 3, Table 2).
b. A decreased GLUT4 expression has been reported in the membrane fraction of the frontal cortex in rats affected by depression and obesity [92], diseases known to have insulin resistance among their comorbidities [93].
c. Activation of GLUT4 by insulin is thought to improve glucose flux into neurons during periods of high metabolic demand, such as during learning or other cognitive tasks [94-96]. If this increased glucose demand is not satisfied, in CM sufferers partly due to brain insulin resistance, and if the brain is unable to effectively utilize ketone bodies [97], as should occur during fasting or carbohydrate restriction, then this would lead to an energy deficit, which would, in turn, trigger a migraine attack [15]. Arnold et colleagues also observed that changes in insulin levels might affect neuronal glucose uptake and metabolism via GLUT4 translocation in response to insulin-IRS1-AKT signalling in the brain regions crucial for cognitive and emotional function [37].
d. A reduced cerebral glucose metabolism and lower ATP concentration in *AD-vulnerable regions* are associated with the severity of peripheral insulin resistance and cognitive impairment [43]. This finding also supports our hypothesis that prolonged peripheral insulin resistance in subjects with chronic migraine leads to a reduced regional cerebral glucose metabolism, which may eventually favour neurodegeneration and the development of AD (Fig. 1).

Moreover, the data from a study carried out on young normal weight women with mild insulin resistance (suffering from polycystic ovary syndrome (PCOS)) strengthens the hypothesis that insulin resistance is, in itself, a primary cause of cerebral glucose hypometabolism. Indeed, a direct association was reported between mild insulin resistance and brain glucose hypometabolism, whatever the degree of overweight or obesity [55]. The same authors observed that women with PCOS had a pattern of reduced regional cerebral glucose metabolism, similar to that observed in the early stages of AD [55]. It was reported that brain regions with low cerebral metabolic glucose rates, e.g., the frontal and parietal cortex show volume reduction [55].

### Alterations in brain mitochondrial bioenergetics

The mitochondrial oxidative phosphorylation system produces most cell energy [98]. Alterations in cellular energy metabolites concentration, such as phosphocreatine (PCr), phosphate (Pi), adenosine diphosphate (ADP), adenosine triphosphate (ATP) and cytosolic free magnesium (Mg2+) suggest abnormal mitochondrial function [59, 70, 80, 99]. Indeed, a reduced PCr/Pi and PCr/ATP ratio indicates an energy deficit. Phosphorus (31P)-magnetic resonance spectroscopy (MRS) can provide information on these metabolites from specific brain regions of interest [83].

As early as 1989 [57, 59, 100, 101], 31P-MRS studies were performed in a variety of migraine subtypes, during either the ictal or the interictal period, mostly focused on the occipital lobe as it was considered the region of interest [82, 83]. These studies [57, 59, 100, 101] were carried out to assess oxidative phosphorylation, i.e., the process responsible for generating 90% of the brain's energy and demonstrated that cerebral cortical energy metabolism is abnormal in migraine. Indeed, the lowest energy metabolites concentrations, compared to controls, were detected in the brain of migraineurs, mostly those with aura, and were associated with reduced glucose metabolism in certain areas such as in the parietal lobe, temporal, occipital and frontal lobe, especially in subjects with CM (studies collected in Table 2).

There is further evidence that altered mitochondrial bioenergetics play a role in migraine pathophysiology [97]. Indeed, neuroimaging studies have shown that ATP and “mitochondrial phosphorylation potential” are decreased in the brain of migraineurs without aura interictally, compared to controls [82]. Moreover, the lowest ATP concentrations were detected in the patients who were most severely affected by migraine [97].

Noteworthy is the fact that some experimental studies on energy metabolism reported that AD and migraine share common brain areas which suffer from reduced glucose metabolism, that also show impaired energy metabolism [43, 70] (Table 2).

In AD, these areas are the temporal, parietal, frontal cortex, and the hippocampus [43, 70, 102]. In migraine, they are temporal, parietal, frontal and occipital lobes (Table 2).

Whilst no genetic association has been reported between mitochondrial DNA in AD and migraine [103]. A large population-based cohort study in Norway reported that mitochondrial genetic variation did not play a major role in migraine pathophysiology [104]. Therefore, based on current evidence, this would be an acquired metabolic disorder of the brain which may be related to insulin resistance.

Growing evidence, mainly from *in vivo* experiments, reported that an altered mitochondrial bioenergetics in the brain occurred in association with brain insulin resistance, with an overproduction of mitochondrial reactive oxygen species, along with mitochondrial depolarization and swelling, and that these two events could lead to the development of cognitive decline and AD [105] (Fig. 2), so that AD is now emerging as type III diabetes mellitus [18].

### Neuroinflammation

Neuroinflammation is considered to be an adaptive response triggered by noxious agents, such as infection, injury and/or tissue stress. It plays a significant role in the pathophysiology of various central nervous system diseases, including migraine and AD [106-110]. There is evidence that inflammation may play a pathophysiological role both before and after the neuronal stress involved in a migraine attack [111, 112].

Firstly, inflammation hinders insulin action. The presence of pro-inflammatory cytokines - tumor necrosis factor-α (TNF-α) and interleukin-6 (IL-6) - leads to a decrease in GLUT4 concentration [113-116]. Moreover, it was reported that nuclear factor-κB (NF-κB) regulates neuroinflammation by increasing oxidative damage and insulin resistance, in experimental diabetic neuropathy [117].

This evidence is in agreement with our “Neuroenergetic hypothesis” [15], which proposes that inflammation may well play a pivotal role in migraine pathophysiology by downregulating GLUT4, increasing brain insulin resistance and, in turn, leading to a reduction in cerebral glucose metabolism and inducing neuronal stress

Indeed, neuronal stress [118] and neurodegeneration, driven by protein misfolding [119], may lead to a neuroinflammatory response in a vicious circle. As early as 2013, Karatas et al. [118] reported, in a migraine animal model, a previously unknown signaling pathway between “stressed neurons” (according to our hypothesis, neurons affected by energy deficiency) and trigeminal afferents during cortical spreading depression (CSD), the presumed cause of migraine aura and headache. CSD is able to trigger NF-κB activation in astrocytes which may link neuronal stress to inflammatory response. Suppression of this cascade by Pannexin-1 channel inhibitor abolishes CSD-induced trigeminovascular activation, dural mast cell degranulation and headache [118].

Therefore, the inflammatory response may occur in parallel with a migraine attack and could play an important role in migraine chronification through trigeminal sensitization, most likely triggered by the release of inflammatory cytokines [111].

Clinical evidence also supports that inflammation plays a role in migraine, as reported by Hagen et al., who carried out a population-based follow-up study on the correlation between high-sensitivity C-reactive protein (hs-CRP) at baseline and the risk of developing migraine 11 years later. They reported that the group with the highest hs-CRP levels had nearly a three-fold higher risk of chronic migraine [120]. In another large-scale population-based study, elevated hs-CRP was associated with headache ≥ 7 days/month, which was particularly evident for those who had migraine with aura [121]. The results of further studies support that migraineurs have higher CRP levels than controls [122, 123].

A recent review on 47 studies analysed cytokines via different mediums and reported persistent alteration in inflammatory regulation in the interictal period in migraine [124].

Interictally, migraine patients have higher interleukin (IL)-1β, IL-6, TNF-α, IL-8, IL-12p70 and CCL3 [125-128] and lower IL-10 levels [125, 128]. Although there are contrasting results, most studies reported a rise in IL-1β, IL-6, and TNF-α [129-132] in the ictal phase.

However, the role of cytokines in migraine is still a question of debate, due to a lack of standardization [124]. Infact, as circadian rhythm influences proinflammatory cytokines levels [133], it makes their determination more challenging.

Sarchielli et al. carried out a study on migraine patients during attacks through serial analysis of internal jugular venous blood samples. They observed a transitory rise in sICAM-1, TNF-α, IL-6 and IL-8 levels and NF-κB activity, along with a transient drop in IκBα expression [134-136], in the first two hours after catheter insertion. The rise in IL-8 lasted until the 4th hour; there was an up-regulation of iNOS from the 4th through the 6th hour which decreased at the end of the attack [134, 136].

Similarly to that observed in migraine, there is a strong link between insulin resistance and neuroinflammation in the pathophysiology of AD [109, 137, 138]. Notably, there is a growing body of research that identifies oxidative stress and neuroinflammation as an early event in the pathogenesis of mild cognitive impairment (MCI) and AD [80].

Chronic low-grade inflammation is associated with poor cognitive performance in the elderly [139]. According to data from recent longitudinal studies, elevated IL-6 levels significantly increase the risk of cognitive decline [140]. Furthermore, a recent meta-analysis of 13 studies reported that high CRP is associated with a higher risk of progression from normal cognition to dementia [141]. Other authors have shown that patients with MCI and AD have higher levels of IL-1β, IL-6 and TNF-α compared to controls [140]. Indeed, some pro-inflammatory cytokines such as TNF-α, IL-6 and IL-12, which are produced peripherally, are able to cross the blood-brain barrier [142, 143]. Their subsequent activation, through the receptor binding, could hinder the insulin effects and promote the disease progression of AD [143].

An important factor in the development of insulin resistance is TNF-α [144]. TNF-α impairs insulin signalling by phosphorylating insulin receptor substrate-1 (IRS-1) and Protein Phosphatase-1 serine [145], therefore acting as an inhibitor of the insulin receptor and phosphatidylinositol-3 kinase signaling [146]. TNF-α also reduces the expression of GLUT4 [116].

Monozygotic twin studies found that 60% of the variation in the production capacity of TNF-α is related to genetic variability [147]. Individuals with the TNF-308G>A polymorphism were classified as high producers [148]. A meta-analysis of 6,682 migraineurs reported that TNF-308G>A polymorphism may be a genetic susceptibility factor for migraine among non-Caucasians [149]. The same polymorphism may be a significant risk factor for AD in East Asians [150]. Therefore, both migraine and AD seem to share a common polymorphism which predisposes the carriers to higher levels of TNF-α, inflammation and most likely insulin resistance.

S100B is a brain-specific protein, also referred to as the “C-reactive protein of the brain*”* [151], produced mainly by astrocytes [151]. S100B stimulates the activity of fructose-1,6-bisphosphate aldolase and of phosphoglucomutase and is able to regulate energy metabolism [151]. Its effects seem to be dose-dependent; at low concentrations it is neuroprotective, whilst at high concentrations it’s able to promote inflammatory activity and to induce apoptosis [152, 153].

Data from *in vitro* experiments reported that the release of S100B by astrocytes can be induced by glucose deprivation [154]. Indeed, recent evidence suggests that S100B is a multifacet pathogenic factor in various neurological disorders, sharing common pathogenic processes that can reasonably be attributed to neuroinflammation [155].

During a migraine attack, sufferers have elevated S100B serum levels [156-159], a marker of glial damage. Moreover, S100B was reported to be significantly elevated during the interictal period [157, 159]. Interestingly, high S100B levels induce glycogen synthase kinase 3beta - dependent hyperphosphorylation of the tau protein which is a hallmark of AD [160]. In mouse models, S100B overexpression exacerbates amyloidosis, accelerating disease progression [161].

Post-mortem studies of AD brains demonstrated a correlation between S100B astrocytic expression and dystrophic neurites in amyloid plaques [162]. S100B was also increased in the temporal lobe, where there is a concentration of neurite plaques in AD patients [163].

**Figure 4.**
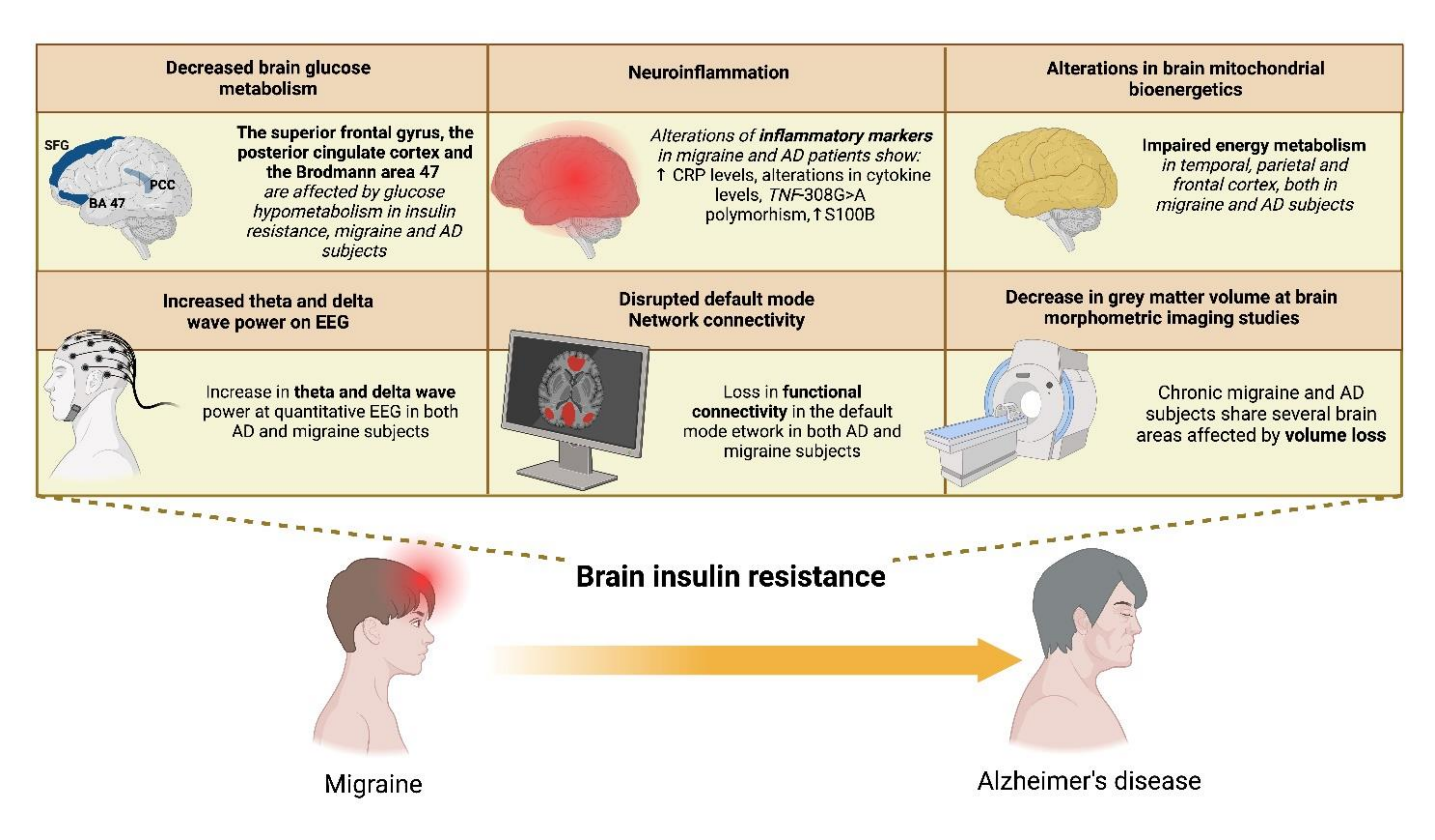
**The intriguing similarities between the pathophysiology of chronic migraine and Alzheimer’s disease (AD)**. Impaired brain glucose metabolism, an alteration in brain mitochondrial bioenergetics and neuroinflammation are common pathophysiological alterations shared by these two pathological conditions that may underlie the reduction in grey matter volume in specific areas, the disrupted default mode network connectivity observed at neuroimaging and the increased theta and delta activity evidenced on EEG in both diseases. Brain insulin resistance may be the pivotal factor linking chronic migraine with AD. PCC, posterior cingulate cortex; SFG, superior frontal gyrus; BA, Brodmann area; AD, Alzheimer’s disease; EEG, electroencephalogram; CRP, C-reactive protein.

Moreover, S100B levels in cerebrospinal fluid, together with other AD biomarkers, such as Aβ and phosphorylated tau, have recently been shown to have an inverse correlation with gray matter volumes and glucose metabolism in key AD-related regions [164, 165]. Interestingly, gene polymorphisms upregulating S100B expression were shown to be associated with an increase in AD risk [165].

However, the precise role neuroinflammation and mitochondrial dysfunction play as contributing factors, in the complex interplay with brain insulin resistance, in both migraine chronification and AD pathologic cascade and clinical progression, is still a question of debate.

## Alterations at neuroimaging and neurophysiological studies: similarities between chronic migraine and Alzheimer’s disease

Interesting similarities have been described by neuroimaging and neurophysiological studies between CM and AD (Table 2), which are reported to be related to the aforementioned common pathophysiological alterations of glucose homeostasis and energy supply to neurons, shared by these two pathological conditions (Fig. 4). Although we are aware that how these changes can be interpreted clinically and implemented into disease management has not yet been defined.

### Decrease in grey matter volume described by brain morphometric imaging studies

In a previous review [15], it was evidenced that insulin resistance is a common factor in several diseases (i.e., CM, major depression, chronic back pain, PCOS, fibromyalgia, osteoarthritis, obesity and T2DM), which are characterized by a reduction in grey matter volume in specific areas: most of these regions (i.e., the frontal/temporoparietal cortex, prefrontal cortex and hippocampus) are affected by volume loss and atrophy also in AD (Table 2), according to magnetic resonance imaging in mice and humans [61, 64, 87].

A correlation analysis revealed that headache frequency was negatively correlated with the volume of the right frontal pole, right lateral orbital gyrus, and medial frontal lobes [76]. Another study [51] reported that there was a focal grey matter decrease in the bilateral anterior cingulate cortex, left amygdala, left parietal operculum, left middle frontal gyrus and inferior frontal gyrus, and bilateral insula in migraineurs compared to controls (Table 2). There was also a statistically significant correlation between a grey matter reduction in the anterior cingulate cortex and the frequency of migraine attacks, in line with the concept that migraine may be a progressive disorder [51].

Schwedt et al. observed that reductions in the regional volume, cortical surface area, and cortical thickness of specific brain regions in the frontotemporal area may distinguish patients with CM from both healthy individuals and patients with episodic migraine with an accuracy of 86% and 84% respectively. Nevertheless, the precision in distinguishing episodic migraine patients from controls was at 67%. This also suggests the existence of a progressive brain structural and metabolic alteration [66].

As previously reported [15], the grey matter volume reduction impacts brain regions that are specifically involved in higher cognitive and affective activities, such as memory, regulation of affective states, emotion, awareness of bodily states and cognitive processing [76, 166-170]. These brain regions are characterized by the expression of insulin-sensitive glucose transporters [37, 171] that optimize the glucose influx into astrocytes and neurons during the aforementioned metabolic high-demand tasks.

As a reduced glucose uptake and glycogen synthesis in astrocytes would impair the neuronal function, it is reasonable to hypothesize that a persistent and progressive brain metabolic alteration (brain insulin resistance and the related disruption of energy supply) would trigger neurodegeneration, altering a signaling cascade and promoting the AD pathologic changes and amyloid-β (Aβ) deposition [44].

### Disrupted default mode network connectivity

The default mode network (DMN), the largest network of functionally correlated brain areas, highly active during rest [172], is crucial for higher cognitive processes, such as memory and executive function. Subjects with dementia show a loss in functional connectivity in the DMN, including the *AD-vulnerable brain regions*: posterior cingulate cortex, prefrontal cortex, lateral temporal cortex, and the hippocampus [43]. Infact, the *AD-vulnerable brain regions* are the first cortical areas to show decreased glucose metabolism in AD patients [43]. Interestingly, persons with T2DM and obese individuals also show diminished functional connectivity within this network. Implying there is an extensive overlap between brain regions affected by AD, T2DM and obesity, especially in regions belonging to the default mode network [43].

Noteworthy is the fact that alterations in the DMN are also detectable in migraine. Indeed, Trufanov et al. demonstrated that patients with CM could be characterized by specific dysfunctional interactions between the DMN and other networks, in the resting state [173].

Russo et al. suggested that DMN abnormalities could represent a prognostic imaging biomarker capable of identifying the migraine patients who are more inclined to migraine chronification [174]. Coppola et al. observed that CM patients had significantly reduced functional connectivity between the DMN than healthy controls [175].

Less functional connectivity and/or lower frequency fluctuations within regions of the DMN have been reported in migraineurs [176, 177]. Kullmann et al. observed that the disruption of functional connectivity and reduced cerebral glucose metabolism in the DMN regions, which are coincident with the *AD-vulnerable brain regions*, is related to the severity of peripheral insulin resistance and cognitive impairment [43]. This seems to provide further evidence in support of our hypothesis that insulin resistance plays a major role in the pathophysiology of CM and AD (Fig. 2).

### Increased theta and delta wave power on EEG

The electrical activity in the brain represents the metabolic state of the neurons and can be investigated and measured by electroencephalography (EEG). An association between hypoglycaemia and changes in the EEG was demonstrated [178], as the EEG is highly sensitive to hypoglycaemic states [179, 180]. There is evidence that hypoglycaemia caused by insulin administration is accompanied by an increase in delta and theta activity [171, 180, 181].

An increase in theta and delta activity was also observed in the topographic EEG mapping of dementia subjects and those with insulin-dependent diabetes mellitus, even when their blood glucose levels were not very low (50-60 mg/dl) [180]. Several older quantitative EEG (qEEG) studies reported increased delta [182, 183] or theta power [182-185] in migraineurs. On the whole, migraineurs had increased relative theta power in all cortical regions and increased delta activity in the painful fronto-central region than controls [186]. Moreover, headache intensity correlated positively with EEG global delta power [186].

Noteworthy is the fact that interictal relative theta power at topographic EEG mapping was higher in migraineurs in the frontocentral regions [186], parieto-occipital regions [184] and temporal regions [183]. Several AD studies have demonstrated that qEEG measurements are able to identify a dysfunction in neuronal (synaptic) activity, in its topographical distribution and synchronization. Infact, generalized EEG slowing, reduced global synchronization and anteriorization of neuronal generators of fast-frequency resting-state EEG activity have been reported in patients along the AD continuum [187]. There is also a strong correlation with qEEG measurements and surrogate markers of AD neuropathology [187].This is supported by a study carried out on amyloid-positive AD patients, where the increase in relative theta power and decrease in relative beta power was reported to be indirect measures of (Aβ-mediated) hyperactivity of pyramidal cells and/or interneuron dysfunction along a pathological continuum [188].

Another study on the baseline EEGs of 18 AD patients used quantitative spectral analysis to investigate the relationship between EEG abnormalities and medial temporal lobe atrophy. There was a statistically significant increase in the power of theta waves in the centro-temporal area in the severe atrophy group. These results suggest that qEEG abnormalities are correlated with the medial temporal lobe atrophy [189]. On the basis of these findings, it is reasonable to hypothesize that a reduced cerebral glucose metabolism is one of the main factors underlying the increase in theta and delta wave power at qEEG in both AD and migraine.

## The brain energy deficit as a driving factor of alzheimer's disease amyloidogenic cascade and protein misfolding

Nowadays, AD is considered to be one of the “protein misfolding diseases”, characterized by protein accumulation, mainly extracellular plaques and intracellular neurofibrillary tangles, which contain the pathological hallmarks of AD, i.e., abnormal Aβ and hyperphosphorylated tau, respectively [190]. As is widely known, aging is the main risk factor for AD. Aging may be linked to AD, at least to some extent, by a perturbed energy metabolism driven by conditions strongly associated with it: insulin resistance, loss of mitochondrial function and a low-grade systemic inflammation [191].

Considering the high energy requirements of basic metabolic processes in neurons, such as protein biosynthesis and folding, it is reasonable that an altered brain glucose metabolism, exacerbated by the time-dependent functional decline, may impair, at least in certain subsets of patients, the proper protein folding and synaptic integrity, leading to neurodegeneration [192] (Figure 2). Indeed, growing evidence supports the hypothesis that insulin resistance and reduced brain glucose metabolism may promote a “shift” towards the amyloidogenic pathway and tau phosphorylation, in the complex interplay of AD pathophysiology [14, 193, 194].

Noteworthy is the fact that there is a close epidemiological relationship between AD and T2DM (81% of cases of AD sufferers had either T2DM or impaired fasting glucose [195]) and that there are striking similarities in the protein misfolding and insulin signalling in both of these diseases, which could be due to the key role that insulin resistance plays in both AD and T2DM [18, 45].

Actually, it has been postulated that insulin resistance is the missing link between brain neuronal loss and pancreatic *β*-cell loss in both diseases, to the extent that some authors, as aforementioned, have called AD “diabetes of the brain” or “type 3 diabetes” [18]. Notably, the hypothesis of an insulin-mediated AD pathology was first conceived by Steen et al. and subsequently developed by other authors [18, 37, 39, 45]. There is recent evidence that insulin has a role in proteostasis, influencing Aβ clearance and tau phosphorylation [14, 18, 44, 193], and that it plays a remarkable, putative role in the development of AD pathological markers [18, 44, 45].

Glycogen synthase kinase-3 remains unphosphorylated and activated in the presence of a reduced insulin stimulation, leading to an impaired glucose metabolism and tau hyperphosphorylation in various metabolic disorders, including AD [196]. In fact, overactivation of glycogen synthase kinase-3 is a common finding in the brains of neurodegenerative patients [196]. Furthermore, β-*N*-acetylglucosamine (GlcNAc)-mediated O-GlcNAcylation has been demonstrated to regulate tau phosphorylation [197]. Interestingly, Liu et al. observed that impaired glucose metabolism downregulates O-GlcNAcylation, consequently leading to tau hyperphosphorylation in an animal model [197]. Other authors demonstrated not only that metabolic stress induces the phosphorylation of endogenous tau but also, remarkably, that tau phosphorylation is reversible upon restoration of the metabolic homeostasis in cell models as well as in a physiological hypometabolic model in vivo [192, 198].

As is widely known, if neuronal glucose metabolism is impaired, as in the case of brain insulin resistance, the oxidative phosphorylation in neurons will be reduced, leading to a decreased ATP production [15]. Interestingly, early research indicated that the inhibition of oxidative phosphorylation causes Aβ precursor protein (βAPP) to transform itself into Aβ, as βAPP can only be partially inserted into synaptic membranes [199]. A series of similar studies strengthened the hypothesis that an abnormal accumulation of Aβ is triggered by oxidative energy metabolism disturbances, which may “switch” the βAPP metabolism towards the amyloidogenic cascade [44, 45, 200-203].

Moreover, acetylcholine deficiency, long recognized as an early functional abnormality in AD, has also been linked to insulin resistance [44, 204]. In fact, acetylcholine transferase, involved in acetylcholine synthesis, is expressed in insulin and insulin-like growth factor-1 receptor-positive cortical neurons [205] and insulin resistance has been linked to a decreased acetylcholine transferase expression and consequently reduced acetylcholine levels in AD [206]. Notably, Aβ oligomers bind to hippocampal neurons and displace insulin receptors from the plasma membrane interrupting normal insulin signaling [207, 208]. Other studies elucidated the detrimental effect of Aβ on mitochondrial metabolism [209] and its potential to induce neuroinflammation [119], putatively triggering a vicious circle, where the metabolic disruption and neurodegeneration enhance each other. Glucose metabolism is also necessary for autophagy, which is responsible for the clearance of folded proteins in the cell so its dysfunction may lead to Aβ aggregation and tauopathy [210].

In summary, a growing body of research clearly indicates that brain regional hypometabolism, which occurs in certain AD brain regions (Figure 3, Table 2) and can be caused by insulin resistance, may hamper a proper proteostasis and facilitate neurodegeneration [192, 198]. Indeed, in agreement with our “Neuroenergetic hypothesis”, aberrant insulin signaling, and energy deficit may well be predisposing metabolic conditions for both the main pathological changes in AD, the Aβ deposition and tau hyperphosphorylation [18, 44, 45, 205] (Figure 2).

## Summary

In summary, considering that:

– Recent meta-analyses demonstrated that migraine is associated with increased risk of all-cause dementia, but in particular that of AD [21-23, 26].
– Altered insulin signalling and glucose homeostasis are frequently observed in both migraine and AD [15, 37, 195].
– Brain insulin resistance is a pathophysiological mechanism widely described in T2DM and AD [18, 37, 39], and has been hypothesized in CM [15].
– An increasing body of evidence supports the hypothesis that brain insulin resistance, reduced cerebral glucose metabolism and the consequent energy deficit may promote a “shift” towards the amyloidogenic pathway and tau phosphorylation [14, 193, 194].
– There are intriguing similarities between the pathophysiology of CM and AD: brain insulin resistance, an impaired brain glucose metabolism, alterations in brain mitochondrial bioenergetics, and neuroinflammation. These seem to be common pathophysiological alterations, underlying a grey matter volume reduction in specific brain areas, a disrupted default mode network connectivity on neuroimaging, and an increased theta and delta activity on EEG, which are shared by these two pathological conditions, i.e., CM and AD.

Based on this evidence, we would like to propose an “extended neuroenergetic hypothesis” (illustrated in Fig. 1 and 2) where brain insulin resistance may be a metabolic bridge that links CM to AD along a pathophysiological continuum.

## Potential Treatments targeting the mechanisms highlighted by the “Neuroenergetic hypothesis”

Although there are significant gaps in the current research in understanding how the mechanisms highlighted at a molecular level can be effectively targeted by new therapies in the clinical practice, we suggest that the aforementioned metabolic abnormalities involving glucose homeostasis, energy deficit and neuroinflammation in the frame of the “Neuroenergetic hypothesis”, may well become appealing targets for preventive therapeutic approaches to both migraine and, possibly, AD, i.e., diet, aerobic exercise and mind-body interventions.

## Diet

An optimal dietary pattern should be able to reduce systemic inflammation [211, 212], exclude high glycemic index foods, be sustainable at long-term and have no adverse effects. The traditional Mediterranean diet is a dietary pattern that meets all four of these requirements [213-215]. In fact, there is evidence supporting [216-218] that diets similar to the traditional Mediterranean one, i.e., the Healthy Eating Plate and the Dietary Approaches to Stop Hypertension (DASH), are efficacious in reducing the frequency and intensity of migraine and its associated disability. Moreover, RCTs, meta-analyses and systematic reviews that, over the past decade, have evaluated the role diet plays in the treatment and prevention of depression [219-221] and dementia [222-227] have suggested that a higher adherence to the Mediterranean diet or similar ones (the DASH, the Healthy Nordic diet and Mediterranean-DASH Intervention for Neurodegenerative Delay) is associated with higher remissions and a lower incidence of depression, slower cognitive decline and a reduction in the risk of developing dementia. Interestingly, a 3-months RCT on the DASH diet in migraine patients reported a decrease in migraine frequency and severity [228]. Accordingly, in another 3-month RCT, a very low-glycemic index diet proved to be as effective as standard pharmacological treatment in migraine prophylaxis [229]. Moreover, a recent 3-year two-arm RCT [230], assessed the effect of a Mediterranean-DASH Intervention for Neurodegenerative Delay and a Mild Caloric Restriction Intervention. Both interventions led to improved overall cognition. Indeed, caloric restriction is a known modulator of insulin signaling in the peripheral tissues and seems to preserve brain energy metabolism during the aging process [231].

Long-term RCTs promoting a Mediterranean diet may be useful to clarify whether improved adherence to such preventive approach may be beneficial in preventing AD or delaying the onset of AD pathological changes and dementia. Some case reports and prospective studies have demonstrated the efficacy of the ketogenic diet for episodic and chronic migraine [232]. This diet mimics, to some extent, the state of fasting and promotes hepatic production of an alternative to glucose as an energy substrate for the brain [97]. This would contribute to the restoration of brain excitability and metabolism and counteracting neuroinflammation in migraine [232].

Moreover, Di Lorenzo et al. investigated the effects of one-month ketogenic diet had on 18 migraneurs. The resulting data demonstrated that not only was there a decrease in the frequency and duration of headache attacks, but also a normalization of some parameters of evoked potentials in response to visual and somatosensory stimuli [233]. Accordingly, Caprio et al., reported on a two month very low-calorie ketogenic diet and demonstrated that it effectively reduced monthly migraine days [234]. Interestingly, it was also proven that the ketogenic diet was able to reduce insulin-resistance in other diseases, like T2DM and PCOS [235, 236].

However, although recent data indicate the possibility of good compliance and an improved quality of life over a span of one year, the safety of a ketogenic diet has not yet been fully assessed in long-term trials [214] and to date, the ketogenic diet does not meet two of the four aforementioned criteria, i.e., the long-term safety and sustainability.

Nevertheless, long interventional trials on the Mediterranean diet such as the Predimed and Cardioprev trial, reported a good 5-year adherence [237, 238]. Moreover, dietary change seemed feasible also in over 70-years olds [239]. However, further studies are needed to support the efficacy and feasibility of a “metabolic” strategy for AD prevention, particularly over the long-term.

## Exercise and Mind-Body interventions

Regular exercise and mind-body interventions are supported by a growing body of evidence as being effective prophylaxis interventions for migraine, AD and age-related cognitive decline. Cross-sectional and population-based studies reported that low physical activity is associated with a higher prevalence of migraine [240]. Regular moderate aerobic physical exercise (>40 min, 3 times per week) seems effective in reducing both the severity and frequency of migraine attacks [241, 242]. Meta-analyses in literature evidence that the frequency, intensity and duration of migraine pain are improved by both strength training and high-intensity aerobic exercise [243, 244]. This effect could be attributed to improved glucose tolerance and increased mitochondrial biogenesis [245, 246]. Two other meta-analyses [247, 248] of prospective studies on physical activity reported that it reduces the risk of dementia and AD in a dose-response fashion [247, 248]. The protective effect observed by these authors may be due to an enhanced hippocampus volume in the more elderly adults, which was induced by physical activity and the ability exercise has to counteract age-related brain volume deterioration [249-251]. A recent clinical study reported that a sedentary lifestyle led to obesity and brain insulin resistance, and that exercise could reverse this metabolic abnormality [46]. This highlights brain insulin resistance as a plausible therapeutic target for the prevention of cognitive decline and dementia due to AD.

Interestingly, according to a recent meta-analysis on MCI patients, mind-body interventions exert a stronger effect on cognitive gain than does exercise alone [252]. Furthermore, in older adults with MCI, mind-body interventions were reported to improve cognitive and everyday function, memory, resilience and mindfulness [253].

In 2021, Wells et al. reported that mindfulness-based stress reduction lessened disability and enhanced the quality of life, well-being and self-efficacy in migraine sufferers. Mindfulness-based stress reduction also mitigated pain catastrophizing and depression and, importantly, lead to a reduction in experimentally induced pain, suggesting a fundamental shift in pain perception and processing [254]. These transversal results are in agreement with research which emphasizes how mind-body interventions can downregulate the expression of pro-inflammatory genes (e.g. NF-κB) [255-259]. Meaning that mind-body interventions could be cost-effective and empowering interventions to target multiple diseases that have an inflammatory basis [260].

Meditation is one of the most common and popular mind-body practices [261].

A 2018 meta-analysis of ten RCTs and 315 migraine patients reported that mindfulness meditation lessens pain intensity [262]. Individuals who engage in long-term meditation practices have numerous neurological benefits. It seems that meditators have an increased cortical thickness, reduced age-related white matter connectivity [263] and atrophy, particularly in the hippocampus, frontal, temporal brain regions and the amygdala [264-267]. This has led to speculation that meditators' brains are less affected by the aging process [268]. An interesting study on a small sample of 6 expert elderly meditators, compared to 67 elder controls, reported that meditators had a higher glucose metabolism than did the at-rest controls in aging-sensitive regions, such as the ventromedial prefrontal and anterior cingulate cortex bilaterally, the right insula, temporoparietal junction and posterior cingulate cortex. It was also reported that expert meditators have more preservation of grey matter volume than controls [269]. Another functional neuroimaging study carried out a network-based analysis of anatomical pathways and observed that the meditators had a stronger connectivity than controls between four areas in the left hemisphere pertaining to the somato-motor, dorsal attention, subcortical and visual networks [270].

Overall, these findings imply that exercise and mind-body interventions are potentially valid, cost-effective and user-friendly ways to counteract age-related disruptions in glucose metabolism ??and reduce the risk of both migraine chronification and cognitive impairment. Ultimately, the multicenter randomized-controlled FINGER (Finnish Geriatric Intervention Study to Prevent Cognitive Impairment and Disability) trial demonstrated that a multimodal intervention, encompassing nutrition, physical activity and cognitive training has the potential to be cost-effective in preventing dementia in the long-term [271].

Although the results at an individual level are rather modest, the societal benefits can be substantial, because of the potentially large target population [271]. This model is now being tested globally [272], by the World-Wide FINGERS Network, and the preliminary results seem to support the hypothesis that the above-mentioned preventive interventions might be beneficial in preventing cognitive decline. However, further clinical studies are mandatory to prove the efficacy of these approaches in reducing the risk of AD.

## CONCLUSIONS

This review highlights a potential missing link between migraine and AD: brain insulin resistance. This is the “core” of our “neuroenergetic hypothesis” and could be a pivotal pathophysiological feature, shared by CM and AD (Fig. 1 and 2).

We describe intriguing similarities between migraine and AD pathophysiology, i.e., a lower cerebral glucose metabolism, alterations in brain mitochondrial bioenergetics and neuroinflammation, all of which most likely underlie the reduction in gray matter volume in specific areas, a disrupted DMN connectivity and the increased theta and delta waves observed at EEG in both migraine and AD (Figure 4). All of these alterations, shared by migraine and AD, could be related, at least partly, to brain insulin resistance, which is bidirectionally related to mitochondrial functional alterations and neuroinflammation in a complex interplay. This prompts us to advocate that brain insulin resistance could be considered a “metabolic bridge” between CM and AD, along a pathophysiological continuum (Figures 1 and 2). Indeed, in the long run, brain insulin resistance and the related energy deficiency might favour the pathological changes characteristic of AD, promoting a shift towards the amyloidogenic cascade and enhancing tau hyperphosphorylation. ??Although further studies are required to support this novel “Neuroenergetic hypothesis” as a bridge linking migraine to AD, hopefully it may elucidate new targets for innovative preventive treatment of these two leading neurological causes of disability worldwide.

## Further research

Further investigations and new approaches are needed for the prophylaxis and treatment of both migraine and AD that, despite extensive efforts, continue to be major causes of disability worldwide. We deem that further research, aimed at completing the puzzle of the “Neuroenergetic hypothesis”, herein presented, should focus on improving our research strategies to investigate brain insulin resistance in the general population. Indeed, this will improve our understanding of how brain insulin resistance relates to chronic migraine and AD.

Furthermore, it would also allow us to clarify whether improving brain insulin resistance could lead to a decrease in headache attacks in chronic migraineurs in a real-world setting. Moreover, it could shed more light on whether improving brain insulin resistance could have any clinically relevant impact on the prevention, or slowing, of AD clinical progression, as has been frequently reported in pre-clinical research [46].

Our hypothesis suggests that treating altered glucose metabolism in episodic migraine sufferers with effective strategies might be a beneficial to prevent the clinical progression to chronic migraine and, maybe, in the long-term, also to reduce the risk of developing AD, although more clinical studies are required to demonstrate this hypothesis and to clarify this still controversial issue [138].

Another welcome line of research, could focus on clarifying the exact mechanisms that could effectively therapeutically target brain insulin resistance at a molecular level, aimed at achieving an indirect reduction of the protein misfolding and/or an improvement in clinical outcomes [47]. It would be also helpful if anti-diabetes drugs, investigated in the context of AD treatment, were also assessed for the treatment of chronic migraine [47]. We are of the opinion that, in the future, long-term RCTs should be carried out to evaluate the cost-effectiveness of lifestyle multimodal interventions (i.e. diet, aerobic exercise and mind-body interventions) as a prophylactic strategy for migraine and AD, as well as RCTs to further evaluate the efficacy and safety of anti-diabetic drugs in the prophylaxis and treatment of chronic migraine and AD [47].
